# GABA and Glutamate Uptake and Metabolism in Retinal Glial (Müller) Cells

**DOI:** 10.3389/fendo.2013.00048

**Published:** 2013-04-17

**Authors:** Andreas Bringmann, Antje Grosche, Thomas Pannicke, Andreas Reichenbach

**Affiliations:** ^1^Department of Ophthalmology and Eye Hospital, Faculty of Medicine, University of LeipzigLeipzig, Germany; ^2^Paul Flechsig Institute of Brain Research, Faculty of Medicine, University of LeipzigLeipzig, Germany

**Keywords:** retina, Müller cells, glutamate, GABA, recycling, retinal pathology

## Abstract

Müller cells, the principal glial cells of the retina, support the synaptic activity by the uptake and metabolization of extracellular neurotransmitters. Müller cells express uptake and exchange systems for various neurotransmitters including glutamate and γ-aminobutyric acid (GABA). Müller cells remove the bulk of extracellular glutamate in the inner retina and contribute to the glutamate clearance around photoreceptor terminals. By the uptake of glutamate, Müller cells are involved in the shaping and termination of the synaptic activity, particularly in the inner retina. Reactive Müller cells are neuroprotective, e.g., by the clearance of excess extracellular glutamate, but may also contribute to neuronal degeneration by a malfunctioning or even reversal of glial glutamate transporters, or by a downregulation of the key enzyme, glutamine synthetase. This review summarizes the present knowledge about the role of Müller cells in the clearance and metabolization of extracellular glutamate and GABA. Some major pathways of GABA and glutamate metabolism in Müller cells are described; these pathways are involved in the glutamate-glutamine cycle of the retina, in the defense against oxidative stress via the production of glutathione, and in the production of substrates for the neuronal energy metabolism.

The vertebrate retina contains two types of neuron-supporting macroglial cells, astrocytes and Müller cells. Astrocytes are restricted to the innermost layers of vascularized retinas (Figure 1). Müller cells are specialized radial glial cells which span the entire thickness of the neural retina (Figure 1). Müller cells support the functioning and metabolism of retinal neurons and are active players in normal retinal function and retinal degeneration (Bringmann et al., 2006). Müller cells provide trophic substances to neurons and remove metabolic waste (Newman, 1994; Tsacopoulos and Magistretti, 1996), mediate the retinal potassium, water, and acid-base homeostasis (Bringmann et al., 2006) and the maintenance of the blood-retinal barrier (Shen et al., 2012), and regulate the retinal blood flow (Metea and Newman, 2006). Müller cells act as living optical fibers which guide light toward the photoreceptors (Franze et al., 2007; Agte et al., 2011). Their processes function as soft, compliant embedding for neurons which supports synaptic plasticity and neurite outgrowth (Lu et al., 2006). Müller cells support the synaptic activity by neurotransmitter recycling, the supply of neurons with precursors of neurotransmitters, and the release of gliotransmitters which affect neuronal activity (Newman, 2004). Müller cells become activated upon virtually all pathogenic stimuli. Reactive Müller cells support the survival of photoreceptors and neurons, but may also contribute to neuronal degeneration (Bringmann et al., 2009). Currently, the many roles of Müller cells in the regulation of retinal function are still not elucidated, and are subject of intensive research.

**Figure 1:**
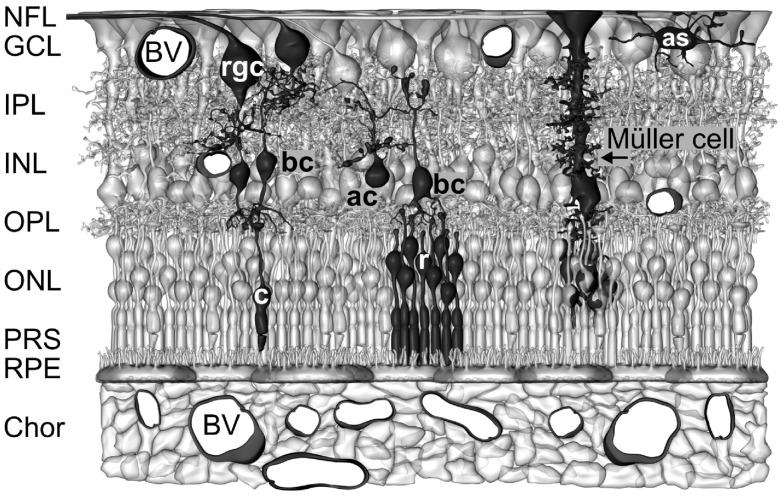
**Müller cells span the entire thickness of the neuroretina**. Schematic drawing of the cellular constituents and basic neuronal circuits of a human retina. The inner retina contact the vitreal cavity (*top*) and contains the following retinal layers: nerve fiber layer (NFL), ganglion cell layer (GCL), inner plexiform layers (IPL), and inner nuclear layer (INL). The outer retina is directed to the outer surface of the eye and contains the following retinal layers: outer plexiform layer (OPL), outer nuclear layer (ONL), the subretinal space containing the photoreceptor segments (PRS), and the retinal pigment epithelium (RPE). The outer retina is supplied with oxygen and nutrients by the blood vessels (BV) in the choroid (Chor), while the inner retina is supplied by intraretinal vessels. Astrocytes (as) are localized in the NFL/GCL. The perikarya of Müller cells are localized in the INL. From the perikaryon, two stem processes of Müller cells extend toward both surfaces of the neuroretina. The funnel-shaped endfeet of Müller cells form (in association with a basement membrane) the inner surface of the retina. In the OPL and IPL, side branches which form perisynaptic membrane sheaths originate at the stem processes. In the ONL, the stem process of Müller cells forms membraneous sheaths which envelop the perikarya of rods (r) and cones (c). Microvilli of Müller cells extend into the subretinal space. ac, amacrine cell; bc, bipolar cell; rgc, retinal ganglion cell.

The precise shaping of synaptic activity depends upon the kinetics of both the presynaptic neurotransmitter release and the re-uptake of the transmitter into the cells. In the retina, photoreceptor cells, neurons, and macroglial cells express high-affinity transporters for neurotransmitters. Müller cells express uptake and exchange systems for various neurotransmitters including glutamate and γ-aminobutyric acid (GABA). This review gives a survey of the present knowledge regarding the involvement of Müller cells in the uptake and metabolism of GABA and glutamate, the relationships between the glial transmitter recycling and the various other functional roles of Müller cells, and the contribution of Müller cell’s transmitter recycling to the neuroprotective and detrimental effects of gliosis.

## GABA Uptake and Metabolism

γ-Aminobutyric acid is the main inhibitory neurotransmitter in the vertebrate retina, used by subclasses of horizontal, amacrine, ganglion, bipolar, and interplexiform cells. The termination of the synaptic action of GABA is achieved by its uptake into presynaptic neuronal terminals and into the surrounding glial cell processes. In addition to neurons such as amacrine and interplexiform cells (Moran et al., 1986; Pow et al., 1996) and, in the fish retina, horizontal and bipolar cells (Nelson et al., 2008), Müller cells and, at least under pathological conditions, astrocytes and microglia take up GABA (Sarthy, 1982; Osborne et al., 1995). It was suggested that in the retinas of most lower vertebrates and birds, GABA removal is almost exclusively mediated by neurons, whereas in the mammalian retina, neurons, and Müller cells remove extracellular GABA (Yazulla, 1986; but, see below). In mammals, GABA is taken up predominantly by amacrine and Müller cells in the inner retina, and almost exclusively by Müller cells in the outer retina (Marc, 1992; but, see below).

### Glial GABA uptake

**Figure 2:**
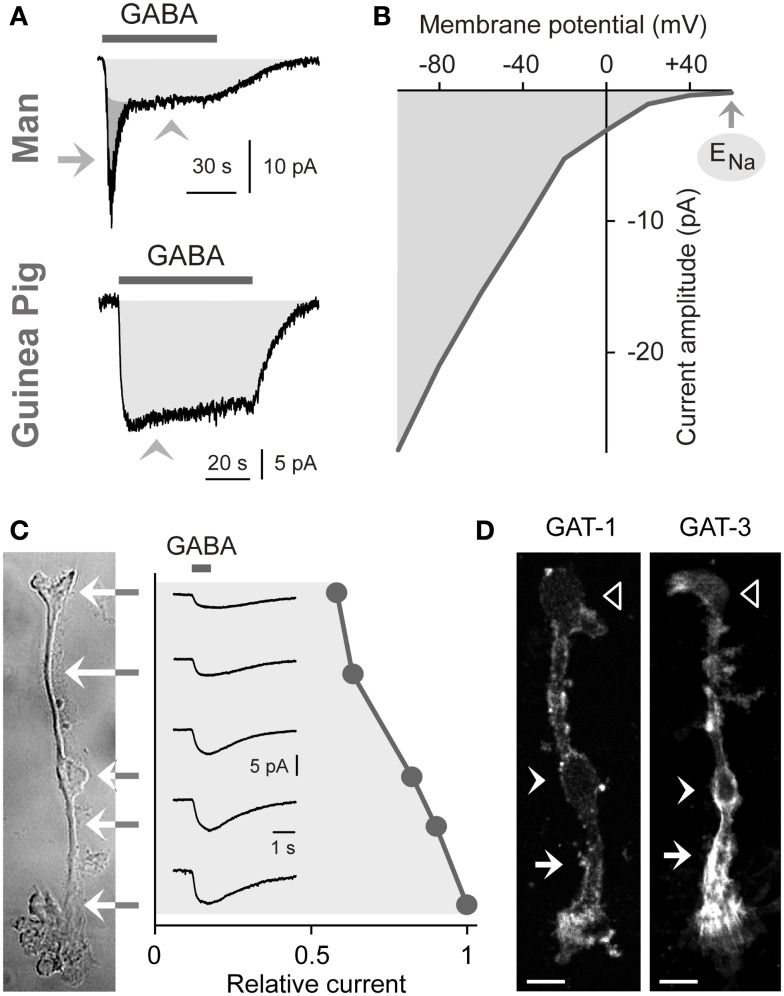
**Electrogenic GABA transport in Müller cells**. The GABA-evoked membrane currents were recorded in freshly isolated Müller cells from man **(A)** and guinea pigs **(A–C)**. **(A)** Extracellular administration of GABA (100 μM) evokes two kinds of inward currents in a human Müller cell: a transient, rapidly inactivating chloride current mediated by GABA_A_ receptors (*arrow*) and a sustained current mediated by electrogenic GABA transporters (*arrowhead*). In guinea pig Müller cells (which lack GABA_A_ receptors), extracellular GABA (1 mM) evokes only a transporter-mediated inward current. The holding potential was −80 mV. **(B)** Current-voltage relation of the GABA transporter currents in guinea pig Müller cells. The amplitude of the transporter currents is zero near the equilibrium potential of sodium ions (E_Na_) (when recorded with symmetrical chloride concentration at both sides of the membrane). GABA was administered at a concentration of 100 μM. **(C)** Subcellular distribution of the GABA transporter currents. The distribution of the currents was determined by focal ejections of GABA (1 mM) onto the following membrane domains of Müller cells: endfoot, inner stem process, soma, inner and outer parts of the outer stem process. **(D)** Distribution of GAT-1 and GAT-3 immunoreactivities in isolated Müller cells of the guinea pig. *Filled arrows*, outer stem process. *Filled arrowheads*, cell soma. *Unfilled arrowheads*, cell endfoot. Bars, 10 μm. With permission from Biedermann et al. (2002, 2004).

The uptake of GABA by Müller cells is mediated by sodium- and chloride-dependent high-affinity GABA transporters (GATs). Per transport step, two sodium ions and one chloride ion are co-transported with one GABA molecule (Qian et al., 1993; Biedermann et al., 2002). The transport process causes inwardly directed membrane currents (Figure 2A) and cellular depolarization. The GAT currents are voltage-dependent. The current amplitude increases (Figure 2B) and the affinity of GABA to the transporter molecules decreases with cellular hyperpolarization (Biedermann et al., 2002). The GAT currents in guinea pig Müller cells display their largest amplitude at the end of the outer stem processes which envelop the terminals and somata of photoreceptor cells (Figure 2C) (Biedermann et al., 2002). GABA at 100 μM is fully cleared from the extracellular space around one Müller cell after 70 ms (Biedermann et al., 2002). Due to this high efficiency of the GABA uptake, Müller cells were suggested to be involved in the rapid termination of the GABAergic transmission in the mammalian retina.

### Expression of GABA transporters

To date four GAT subtypes have been described (GAT1-4) in addition to the vesicular transporter (VGAT) (Schousboe, 2003). The expression of GAT subtypes in Müller cells varies among the species. Müller cells of the guinea pig express GAT-1 and GAT-3 (Biedermann et al., 2002). The transporter proteins show elevated expression in the outer stem process of the cells (Figure 2D) (Biedermann et al., 2002). Müller cells of the chick and rat also express GAT-1 and GAT-3 (Brecha and Weigmann, 1994; Honda et al., 1995; Johnson et al., 1996) while Müller cells of the rabbit express GAT-3 (Hu et al., 1999). On the other hand, Müller cells of the bullfrog express GAT-1 and GAT-2 (Zhao et al., 2000), and Müller cells from other lower vertebrates such as tiger salamander and salmon apparently do not express GAT proteins (Yang et al., 1997; Ekström and Anzelius, 1998).

Cultured avian Müller cells, but not avian Müller cells *in situ*, accumulate GABA (Marshall and Voaden, 1974; Pow et al., 1996; Calaza et al., 2001). However, a failure in demonstrable GABA uptake in Müller cells of distinct lower vertebrates and birds should be considered with caution. It can not be ruled out that GABA is rapidly converted by the GABA transaminase in Müller cells, resulting in a lack of detectable GABA in Müller cells. It has been shown, for example, that turtle Müller cells display a very little GABA transport activity but high levels of GABA transaminase (Sarthy and Lam, 1978).

Whether or not neuronal cells participate in the GABA clearance within the outer mammalian retina is unclear and may depend on the species investigated. Horizontal cells of guinea pigs and mice do not express GATs (Guo et al., 2010; Deniz et al., 2011) while rod bipolar and horizontal cells of monkeys express GAT-3 (Lassová et al., 2010).

### GABA metabolism

**Figure 3:**
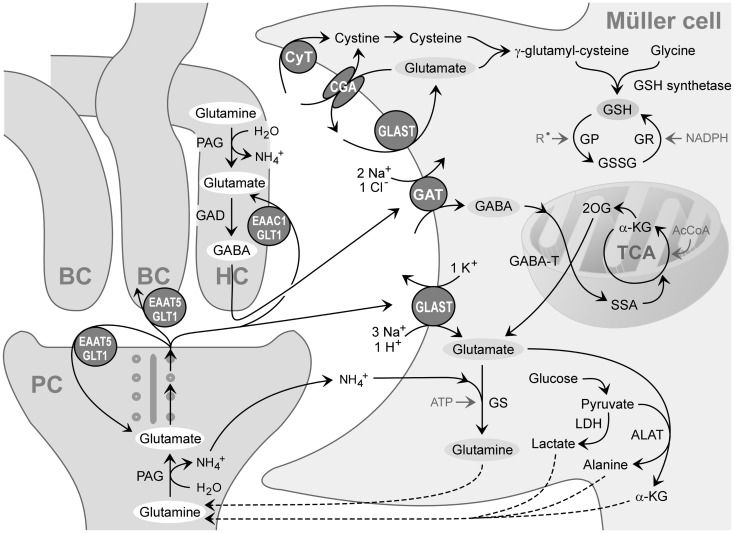
**Recycling of amino acid neurotransmitters in the outer plexiform (synaptic) layer of the mammalian retina**. The ribbon synapse of a photoreceptor cell (PC) synthesizes glutamate which is continuously released during darkness. The postsynaptic elements are dendrites of bipolar (BC) and horizontal cells (HC). Horizontal cells release GABA which is formed from glutamate. The synaptic complexes are surrounded by Müller cell sheets; the right side shows neurotransmitter uptake systems and some metabolization ways of Müller cells. Glutamate, GABA, and ammonia (${\text{NH}}_{\text{4}}^{\text{+}}$) are transported into the Müller cell and transformed to glutamine and α-ketoglutarate (α-KG). Glutamine is released from Müller cells and serves as precursor for the transmitter synthesis in neurons (glutamate-glutamine cycle). Lactate, alanine, pyruvate, α-ketoglutarate, and glutamine are utilized by neurons as substrates for their energy metabolism. The mitochondrial enzyme GABA transaminase (GABA-T) catalyzes the formation of glutamate from 2-oxoglutarate (2OG), coupled to a conversion of GABA to succinate semialdehyde (SSA). Another metabolic way is the production of reduced glutathione (GSH) which is an intracellular antioxidant, released from Müller cells and taken up by neurons under oxidative stress conditions. AcCoA, acetyl coenzyme A; ALAT, alanine aminotransferase; CGA, cystine-glutamate antiporter; CyT, cystine transporter; EAAC1, excitatory amino acid carrier 1; EAAT5, excitatory amino acid transporter 5; GAD, glutamic acid decarboxylase; GAT, GABA transporter; GLAST, glutamate-aspartate transporter; GLT-1, glutamate transporter-1; GP, glutathione peroxidase; GR, glutathione reductase; GS, glutamine synthetase; GSSG, glutathione disulfide; LDH, lactate dehydrogenase; PAG, phosphate-activated glutaminase; $R^{∙}$, free radicals; TCA, tricarboxylic acid cycle.

When GABA enters the Müller cell interior, it is metabolized by the mitochondrial enzyme GABA transaminase which catalyzes the formation of glutamate from 2-oxoglutarate, coupled to a conversion of GABA to succinate semialdehyde (Figure 3). Due to the efficiency of the GABA transaminase reaction, Müller cells display a very low level of intracellular GABA (Marc et al., 1998a). Under diabetic and ischemic conditions, GABA rapidly accumulates in Müller cells (Ishikawa et al., 1996; Napper et al., 2001) due to a decrease in the GABA transaminase activity (Barnett and Osborne, 1995; Ishikawa et al., 1996).

## Glutamate Uptake and Metabolism

### Glutamate uptake

Glutamate is the main excitatory neurotransmitter in the retina, used in the forward transmission of visual signals by photoreceptors, bipolar, and ganglion cells. In the outer retina, glutamate is continuously released from photoreceptor terminals in the dark; this release is inhibited by light. In the inner plexiform layer, ON-bipolar cells release glutamate during light exposure whereas OFF-bipolar cells release glutamate in the dark.

#### Importance of glial glutamate uptake

In the neural retina, photoreceptors, neurons, and macroglial cells express high-affinity glutamate transporters (GLT) (Rauen and Wiessner, 2000). In the inner retina, Müller cells are responsible for the bulk of glutamate uptake (White and Neal, 1976; Harada et al., 1998). By their glutamate uptake, Müller cells are involved in setting the signal-to-noise ratio of synaptic transmission and the spatial resolution of light-induced signaling. Under pathological conditions, when the transport into Müller cells is reduced, more glutamate is transported into inner retinal neurons (Barnett et al., 2001; Holcombe et al., 2008). In the outer retina, the bulk of glutamate released from photoreceptor terminals was suggested to be removed by presynaptic transporters of photoreceptor cells (Hasegawa et al., 2006) and possibly by postsynaptic transporters at horizontal and bipolar cells (Rauen et al., 1996). Here, Müller cells take up glutamate which diffuses out of the synaptic clefts; this prevents the lateral spread of the transmitter and ensures visual resolution (Rauen et al., 1996). However, a recent study showed that inhibition of the main GLT of Müller cells (GLAST; see below) is highly effective in blocking the synaptic transmission in the outer plexiform layer, where photoreceptor terminals are localized (Figure 1) and in inducing a permanent electroretinogram deficit, whereas inhibition of GLT-1 (see below) caused no permanent electroretinogram changes (Levinger et al., 2012).

The glutamate uptake by Müller cells in the inner retina contributes to the rapid termination of the postsynaptic action of glutamate in non-spiking retinal neurons and in ganglion cells (Matsui et al., 1999; Higgs and Lukasiewicz, 2002). Here, Müller cell-provided glutamate uptake is an active player in synaptic transmission. When the retinal glutamate transport was blocked, the amplitude and duration of ganglion cell EPSCs increased dramatically whereas when only the neuronal transport was blocked, little change in synaptic currents was observed (Higgs and Lukasiewicz, 2002).

There are further data supporting the assumption that glutamate uptake and metabolism by Müller cells is more directly involved in the regulation of the activity of inner retinal neurons than that of photoreceptors. For example, the precursor of the glutamate synthesis in bipolar and ganglion cells, glutamine, is derived almost exclusively from Müller cells whereas photoreceptor cells synthesize only a part of their glutamate from Müller cell-derived glutamine (Pow and Robinson, 1994) while the other parts are derived from the re-uptake by the presynapse (Hasegawa et al., 2006) and, possibly, the transamination of α-ketoglutarate (Pow and Robinson, 1994). In addition, a significant amount of GABA in amacrine cells is synthesized from glutamate after uptake of Müller cell-derived glutamine (Pow and Robinson, 1994).

The clearance of synaptic glutamate by Müller cells is required for the prevention of neurotoxicity. After experimental inhibition of the glial glutamate uptake, even low concentrations of extracellular glutamate became neurotoxic (Izumi et al., 1999). Alterations in the activity of glial GLTs might be also involved in the regulation of the glial support of the neuronal signal transfer from photoreceptors to retinal ganglion cells. Repetitive light stimulation of the guinea pig retina induces a glial calcium response that occurs simultaneously throughout the whole length of Müller cells (Rillich et al., 2009). This response is induced by photoreceptor-to-glia signaling which evokes a hyperpolarization-induced influx of extracellular calcium (Rillich et al., 2009). The hyperpolarization is induced by decreases in the subretinal potassium and in the activity of electrogenic GLTs due to the light-induced reduction in photoreceptor activity and by the action of zinc ions (Rillich et al., 2009) which are released from photoreceptors (Redenti et al., 2007) and which directly inhibit GLTs (Spiridon et al., 1998). The glial forward signaling may include the opening of calcium-dependent potassium channels to increase the potassium buffering capacity, and a calcium-dependent release of glutamate to prevent hypoosmotic Müller cell swelling (Wurm et al., 2011; Slezak et al., 2012). Thus, alterations in the activity of glial GLTs may regulate glial neuron-supporting functions via, among other mechanisms, regulation of the membrane potential of Müller cells.

#### Glial glutamate transporters

Müller cells regulate retinal glutamate levels via sodium-dependent and -independent uptake systems (Sarthy et al., 2005). The sodium-dependent uptake involves at least five excitatory amino acid transporters (EAAT1-5) (Kanai and Hediger, 2004). EAATs mediate the transport of l-glutamate, l-aspartate, and d-aspartate. The major GLT of Müller cells is the electrogenic, sodium-dependent, high-affinity glutamate-aspartate transporter (GLAST or EAAT1) (Rauen, 2000). In Müller cells of the mouse, approximately 50% of glutamate is taken up via GLAST, another 40% through electroneutral, sodium-dependent GLTs, and 10% via sodium-independent transporters or exchangers (Sarthy et al., 2005). The presence of further EAATs in Müller cells of various species has been described: glutamate transporter-1 (GLT-1 or EAAT2; goldfish, rat, man), excitatory amino acid carrier 1 (EAAC1 or EAAT3; carp, bullfrog, rat, man), EAAT4 (rat, cat), and EAAT5 (rat) (Rauen, 2000; Vandenbranden et al., 2000; Zhao and Yang, 2001; Kugler and Beyer, 2003; Fyk-Kolodziej et al., 2004; Ward et al., 2005).

Rat Müller cells express, in addition to normally spliced GLAST, the splice variants GLAST1a and 1b which lack exon 3 and 9, respectively (Macnab et al., 2006; Macnab and Pow, 2007). While GLAST is localized throughout the Müller cell membrane, GLAST1a is localized preferentially to the endfeet and inner stem processes, suggesting a selective regulation of GLAST function in different membrane domains of the cells (Macnab et al., 2006).

Glutamate-aspartate transporter is essential for the maintenance of normal synaptic transmission. Knockout or antisense knockdown of GLAST results in a marked suppression of the electroretinogram b-wave, which reflects the depolarization of ON-bipolar cells, and oscillatory potentials, whereas GLT-1 knockout mice exhibit minimal compromise of retinal function (Harada et al., 1998; Barnett and Pow, 2000). In GLAST knockout mice, the total retinal levels of glutamate and GABA, which is produced from glutamate (Figure 3), is increased about twofold compared to that in the wild-type (Sarthy et al., 2004). While the retinas of GLAST and GLT-1 knockout mice show a benign phenotype, retinal injury after ischemia is exacerbated, suggesting that both transporters play a neuroprotective role (Harada et al., 1998).

#### Ion dependency of the glial electrogenic glutamate transport

**Figure 4:**
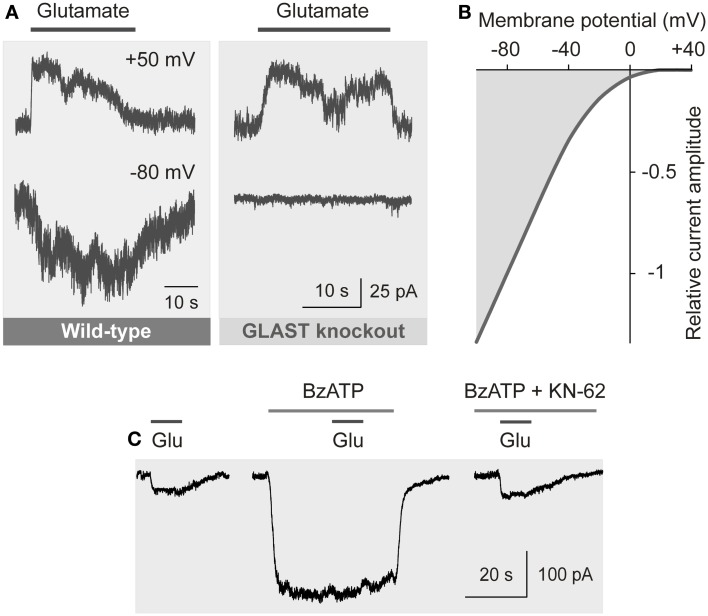
**Electrogenic glutamate transport in Müller cells**. Whole-cell records of membrane currents were made in freshly isolated cells. **(A)** Administration of glutamate (1 mM) to a Müller cell of a wild-type mouse evoked inward currents at −80 mV, and outward currents at +50 mV. The inward currents were mediated by the sodium-dependent glutamate uptake, while the outward currents were mediated by the anionic conductance of the glutamate transporter. (The amplitude of the anionic conductance was increased by replacing extracellular chloride with thiocyanate.) In a cell of a GLAST knockout mouse, glutamate did not evoke inward currents, whereas outward currents remained. The absence of inward currents may suggest that at the resting membrane potential of murine Müller cells the electrogenic glutamate uptake is mediated by GLAST. The presence of the chloride conductance may suggest that EAAT5 (which has a large chloride conductance and minimal glutamate transport capability) is also expressed in the cells. **(B)** Current-voltage relation of the glutamate transporter currents in Müller cells of the guinea pig. (The anionic outward conductance is minimal because extracellular chloride instead of thiocyanate was used used to record the currents.) **(C)** Activation of ionotropic P2X_7_ receptors (which mediate cation currents and thus a depolarization of the cells) decreases the electrogenic uptake of glutamate in human Müller cells. The uptake currents evoked by glutamate (Glu; 100 μM) were diminished in the presence of the P2X_7_ receptor agonist 2′-/3′-*O*-(4-benzoylbenzoyl)-ATP (BzATP; 10 μM). Inhibition of P2X_7_ activation by KN-62 (1 μM) suppressed the BzATP-evoked current (and cell depolarization), resulting in glutamate uptake currents similar in amplitude as under control conditions. With permission from The Society for Neuroscience (Pannicke et al., 2000) and Sarthy et al. (2005).

The transport of glutamate by EAATs involves the co-transport of three sodium ions and one proton, and the counter-transport of one potassium ion, with each glutamate anion (Kanai and Hediger, 2004). The coupling of the glutamate transport with ion transport allows a transport of glutamate into cells against a concentration gradient. The transport of sodium ions into the cell generates inward currents (Figure 4A) and cellular depolarization (Brew and Attwell, 1987; Barbour et al., 1988).

The amplitude of the electrogenic glutamate transport is voltage-dependent (Figure 4B); a very negative membrane potential is essential for the efficient uptake of glutamate (Brew and Attwell, 1987). Cell depolarization, for example by increases in extracellular potassium as occurring in ischemia and glaucoma, substantially decreases the uptake rate. EAATs are GLTs and chloride channels (Figure 4A) (Ryan et al., 2004). The chloride conductance of GLAST is relatively low when compared to EAAT4/5 (Grewer and Rauen, 2005). The glutamate-induced anion conductance observed in Müller cells from GLAST knockout mice (Figure 4A) might be mediated by EAAT5 (Sarthy et al., 2005).

#### Reversal of glial glutamate transport

Under conditions of severe depolarization, Müller cells release glutamate via reversal of electrogenic GLTs (Szatkowski et al., 1990; Billups and Attwell, 1996). The transporter-mediated release of glutamate from Müller cells may contribute to excitotoxic damage to neurons (Szatkowski et al., 1990; Billups and Attwell, 1996; Maguire et al., 1998). Müller cells may also release aspartate, an agonist at *N*-methyl-d-aspartate (NMDA) receptors, via reversal of GLTs; glia-derived aspartate contributes to the activation of NMDA receptors during ischemia (Marcaggi et al., 2005).

#### Cystine-glutamate antiport

Another mechanism of non-vesicular glutamate release is the transport via the electroneutral, sodium-independent, and chloride-dependent cystine-glutamate antiporter (Kato et al., 1993). This antiporter mediates an uptake of cystine in exchange for glutamate; cystine is used for the production of the antioxidant glutathione (Figure 3) (see below). Because this antiporter transports cystine using the transmembrane gradient of glutamate as driving force (Bannai and Tateishi, 1986), the exchanger can also mediate an uptake of glutamate when the extracellular concentration of glutamate is high. Oxidative stress induces increased expression of the antiporter (Mysona et al., 2009). This antiporter may contribute to the release of glutamate from Müller cells under oxidative stress conditions when an elevated production of glutathione and, therefore, an increased uptake of cystine is required (Kato et al., 1993).

#### Vesicular release of glutamate

After its uptake in Müller cells, glutamate can be also concentrated in secretory vesicles. Müller cells of adult mice release glutamate by a calcium-dependent non-vesicular mechanism and by calcium-dependent exocytosis of glutamate-containing vesicles (Slezak et al., 2012). Müller cell-derived glutamate is part of an autocrine glutamatergic-purinergic signaling cascade that prevents osmotic swelling of the cells (Wurm et al., 2011; Slezak et al., 2012). Glutamate released from Müller cells may also modify the light-induced neuronal activity (Newman and Zahs, 1998).

#### Regulation of GLAST

The expression and activity of GLAST in Müller cells is regulated by the availability of glutamate. Extracellular glutamate increases the expression of GLAST (Taylor et al., 2003) while extended exposure to high glutamate induces a time-dependent internalization of the transporters (Gadea et al., 2004). Activation of protein kinase C, e.g., after activation of ionotropic glutamate receptors or of mGluR1 and mGluR5 (Lopez-Colome et al., 1993; Lopez et al., 1998), may decrease the glutamate uptake activity (Bull and Barnett, 2002). An enhanced expression or activity of GLAST in Müller cells was observed under certain pathological conditions (Reichelt et al., 1997a) such as ischemia (Otori et al., 1994). Further factors that increase GLAST expression are cyclic AMP (Sakai et al., 2006) and neurotrophic factors such as glial cell line-derived neurotrophic factor, brain-derived neurotrophic factor (BDNF), and pigment epithelium-derived factor (PEDF) (Delyfer et al., 2005a; Dai et al., 2012; Xie et al., 2012).

#### Glutamate uptake – pathology

Elevated extracellular glutamate causes neuronal loss in many retinal disorders including glaucoma, ischemia, diabetic retinopathy, and inherited photoreceptor degeneration (Lieth et al., 1998; Dkhissi et al., 1999; Delyfer et al., 2005b). The uptake of glutamate by Müller cells blocks the neurotoxic effect of the transmitter (Izumi et al., 1999). A malfunction of the glial glutamate transport thus contributes to the increase in extracellular glutamate up to excitotoxic levels. Retinal ischemia and diabetes do not significantly alter the expression of GLAST (Barnett et al., 2001; Pannicke et al., 2005, 2006; Ward et al., 2005) but reduce the efficiency of the glutamate transport into Müller cells (Barnett et al., 2001; Li and Puro, 2002). Under these conditions, a high amount of glutamate is transported into photoreceptor, bipolar, and ganglion cells (Barnett et al., 2001).

Experimental glaucoma causes a failure of GLAST activity that results in a decreased accumulation of glutamate in Müller cells and in a significant glutamate uptake by retinal ganglion cells; the failure of GLAST coincides with the excitotoxic damage to the retina (Holcombe et al., 2008). Deletion of GLAST in mice leads to optic nerve degeneration similar to normal tension glaucoma (Harada et al., 2007). Increased intraocular pressure causes retinal hypoxia that stimulates free radical formation in the mitochondria and lipid peroxidation which disrupts the glutamate transport into Müller cells. A similar mechanism, i.e., malfunction of the glutamate transport into Müller cells caused by free radicals formed in mitochondria, may explain the retinal ganglion cell death in Leber hereditary optic neuropathy (Beretta et al., 2006).

A major factor that decreases the efficiency of the electrogenic glutamate transport is the depolarization of Müller cells (see above) (Napper et al., 1999). Depolarization of Müller cells can be induced by inflammatory lipids such as arachidonic acid and prostaglandins which are produced under oxidative stress conditions (Birkle and Bazan, 1989; Landino et al., 1996). These inflammatory mediators inhibit the sodium-potassium-ATPase, resulting in cellular depolarization (Lees, 1991; Staub et al., 1994). Arachidonic acid directly inhibits also the electrogenic GLTs (Barbour et al., 1989). Increased membrane localization of the sodium-potassium-ATPase, as induced by interleukin-1 (Namekata et al., 2008), may counterregulate the decrease in glutamate uptake into Müller cells.

Efficient glutamate uptake by Müller cells depends on the very negative membrane potential, around −80 mV, constituted by ample expression of inwardly rectifying potassium (Kir) channels (Kofuji et al., 2000). Human Müller cells of patients with various retinopathies such as retinal detachment, proliferative vitreoretinopathy, diabetic retinopathy, and glaucoma display a depolarization as consequence of a functional inactivation or downregulation of Kir channels (Reichelt et al., 1997a). Inactivation of Kir channels and depolarization of Müller cells were also observed in animal models of various retinopathies including retinal detachment, ischemia-reperfusion, and diabetic retinopathy (Francke et al., 2001; Pannicke et al., 2004, 2006). On the other hand, human Müller cells from patients with the above-mentioned retinopathies display an increase in the density of the GLT currents when compared to cells from donors (Reichelt et al., 1997a). An increase in GLAST labeling was also observed in experimental retinal detachment (Sakai et al., 2001). The increased expression of GLTs may represent a counterregulation in response to long-lasting cellular depolarization. Chick and human Müller cells express P2X_7_; activation of this receptor, e.g., by ATP released from Müller cells (Newman, 2003; Reichenbach and Bringmann, 2013), causes membrane depolarization which impairs the uptake of glutamate (Figure 4C) (Pannicke et al., 2000; Anccasi et al., 2013). High-glucose was shown to induce downregulation of GLAST and Kir channels in cultured Müller cells (Zeng et al., 2010; Xie et al., 2012; but, see Mysona et al., 2009). Treatment with PEDF or taurine inhibited these effects (Zeng et al., 2010; Xie et al., 2012). Dietary taurine ameliorates diabetic retinopathy in part via increased GLAST expression (Zeng et al., 2009). Further factors which reduce the glutamate transport in Müller cells are a reduction of the extracellular pH, as occurring in ischemia, zinc ions released from photoreceptors, and an increase in intracellular glutamate as observed after retinal detachment and in experimental diabetic retinopathy (Billups and Attwell, 1996; Marc et al., 1998b; Spiridon et al., 1998; Gowda et al., 2011).

#### Uptake of ammonia

Ammonia is formed in glutamatergic and GABAergic neurons in the course of the generation of glutamate from glutamine (Figure 3). It is released and taken up by glial cells (Tsacopoulos et al., 1997). Ammonia induces an alkalinization of the Müller cell interior which stimulates the glutamate uptake through GLAST. The mechanism of ammonia uptake by Müller cells is unclear. Retinal glial cells of the bee take up ammonia via a chloride cotransporter (Marcaggi et al., 2004).

### Glutamate metabolism

#### Production of glutamine

After being taken up by Müller cells, glutamate is amidated to glutamine by the enzyme, glutamine synthetase (Figure 3). Glutamine can be also transaminated to α-ketoglutarate which is released and taken up by neurons as a substrate for their oxidative metabolism, utilized for the production of glutathione, or loaded into secretory vesicles (Figure 3). In the neural retina, glutamine synthetase is localized in the cytosol of astrocytes and Müller cells (Riepe and Norenburg, 1977). Glutamine is released from Müller cells and taken up by neurons as a precursor for the synthesis of glutamate and GABA (Figure 3) (Pow and Crook, 1996). Alternatively, glutamine is transported into the mitochondria of Müller cells (see below). The shuttle of glutamate and glutamine, respectively, between neurons and Müller cells is known as glutamate-glutamine cycle.

The glutamine synthetase activity enhances the rate of the glutamate uptake by Müller cells (Shaked et al., 2002). Due to the efficiency of the glutamine synthetase, free glutamate in Müller cells can be demonstrated immunohistochemically only when the glutamine synthetase activity is inhibited pharmacologically or under pathological conditions (Pow and Robinson, 1994; Marc et al., 1998b). When the glutamine synthetase in Müller cells is pharmacologically blocked, bipolar and ganglion cells lose their free glutamate content, and the animals become rapidly, i.e., within 2 min, functionally blind (Pow and Robinson, 1994; Barnett et al., 2000). The lack of immunohistochemically detectable free glutamate in bipolar and ganglion cells after inhibition of the glutamine synthetase suggests that these neurons do not synthesize significant amounts of glutamate from other substrates than glutamine (Pow and Robinson, 1994). On the other hand, inhibition of the glutamine synthesis decreases, but not abolishes, the level of detectable free glutamate in photoreceptor cells (Pow and Robinson, 1994). This suggests that photoreceptor cells take up significant amounts of glutamate from the synaptic cleft (Hasegawa et al., 2006) and are able to synthesize glutamate by transamination of α-ketoglutarate (Pow and Robinson, 1994).

Müller cells possess enzymes that are involved in the *de novo* synthesis of glutamate from pyruvate, e.g., pyruvate carboxylase, that catalyzes the carboxylation of pyruvate to oxaloacetate as substrate of the Krebs cycle, and glutamate dehydrogenase, that converts α-ketoglutarate to glutamate (Gebhard, 1992; Ola et al., 2011a). Glutamate dehydrogenase is able to metabolize glutamate at relatively low pH (Zaganas et al., 2012) that prevails in glial cells following glutamate uptake (Bouvier et al., 1992). The activity of the malate-aspartate shuttle in Müller cells is low (LaNoue et al., 2001) due to the low expression of the aspartate aminotransferase (Gebhard, 1991) and of glutamate-aspartate exchangers (Xu et al., 2007). Thus, the bulk of free glutamate is converted to glutamine, and only a small fraction of glutamate is transported into the mitochondria (Poitry et al., 2000). However, under pathological conditions, when the expression of glutamine synthetase is decreased (see below), more glutamate enters the mitochondria of Müller cells. The loss of the glucocorticoid-mediated inhibition of the expression of the glutamate-aspartate exchanger (Ola et al., 2005) under such conditions (see below) may increase the importance of oxidative glutamate metabolism.

#### Regulation of the glutamine synthetase

The gene transcription of both GLAST and glutamine synthetase is stimulated by glucocorticoids (Gorovits et al., 1996). The upstream region of the glutamine synthetase gene contains a glucocorticoid response element (GRE) that can bind the glucocorticoid receptor protein (Zhang and Young, 1991). There is an inverse relation between the expression of glutamine synthetase and Müller cell proliferation in the developing and injured mature retina (Gorovits et al., 1996; Kruchkova et al., 2001). At early developmental stages, the c-Jun protein, which is a component of the AP1 complex of transcription factors that regulates cellular proliferation, is abundant in proliferating retinal cells. This protein renders the glucocorticoid receptor molecules transcriptionally inactive, and glucocorticoids cannot induce the expression of glutamine synthetase (Berko-Flint et al., 1994). Concomitant with a decline in cell proliferation and c-Jun expression, the developing retina acquires the capability to express glutamine synthetase in response to glucocorticoids.

#### Glutamine synthetase – pathology

The expression of the glutamine synthetase is regulated by glutamate. The expression of glutamine synthetase in Müller cells is reduced when the major glutamate-releasing neuronal population, the photoreceptors, degenerate, as observed in inherited photoreceptor degeneration, retinal light injury, and retinal detachment (Lewis et al., 1989; Grosche et al., 1995; Härtig et al., 1995). A decline in glutamine synthetase expression and activity was also observed under ischemic, inflammatory, and traumatic conditions, and in glaucoma (Nishiyama et al., 2000; Kruchkova et al., 2001; Moreno et al., 2005). Downregulation of the glutamine synthetase results in a depletion of neuronal glutamate (Gionfriddo et al., 2009). No alterations, or even a slight enhancement, in the glutamine synthetase expression in Müller cells was observed in diabetic retinopathy and after optic nerve crush (Mizutani et al., 1998; Lo et al., 2001; Chen and Weber, 2002; Gerhardinger et al., 2005; but, see Yu et al., 2009). An increase in the glutamine synthetase expression was also observed under conditions of increased ammonia (Germer et al., 1997; see below).

Downregulation of the glutamine synthetase in the rat retina by using siRNA induces glial dysfunction which results in a breakdown of the blood-retinal barrier (Shen et al., 2010). This suggests that impairment of Müller cell’s glutamate metabolism also disturbs the integrity of the blood-retinal barrier.

#### Regulation of glutamine synthetase by soluble factors

The decline in the glutamine synthetase expression in Müller cells under pathological conditions is induced, at least in part, by soluble factors such as basic fibroblast growth factor (bFGF) and interleukin-1ß (Kruchkova et al., 2001; Shen and Xu, 2009). These factors increase the level of c-Jun and inhibit the glucocorticoid-induced expression of the glutamine synthetase (Kruchkova et al., 2001; Shen and Xu, 2009). bFGF is rapidly released in the retina after detachment (Geller et al., 2001), and increasingly expressed under ischemic and various other pathological conditions (Miyashiro et al., 1988; Gao and Hollyfield, 1996; Kruchkova et al., 2001). Though bFGF is a major neurotrophic factor which supports neuronal survival (Faktorovich et al., 1990), the bFGF-induced downregulation of the glutamine synthetase might rather aggravate neuronal degeneration. The decrease in glutamine synthetase expression after retinal detachment is likely also a result of the interruption in the supply with PEDF after the separation of Müller cells from the pigment epithelium (Jablonski et al., 2001). PEDF also inhibits the interleukin-1ß-induced downregulation of the glutamine synthetase in diabetic retinopathy (Shen et al., 2011). In addition to PEDF, hydrocortisone, taurine, and BDNF increase the expression of glutamine synthetase (Zeng et al., 2010; Ola et al., 2011b; Dai et al., 2012). Cannabidiol preserves the glutamine synthetase activity by blocking tyrosine nitration that inhibits the enzyme in diabetes (El-Remessy et al., 2010).

#### Ammonia-induced regulation of glutamine synthetase – hepatic retinopathy

The expression of glutamine synthetase is also regulated by the availability of ammonia. Elevated ammonia induces upregulation of the enzyme (Reichenbach et al., 1995b; Germer et al., 1997). As the glutamine synthetase is the most important enzyme available for ammonia detoxification in the retina (Figure 3), this is an important additional function of neurotransmitter recycling by Müller cells.

**Figure 5:**
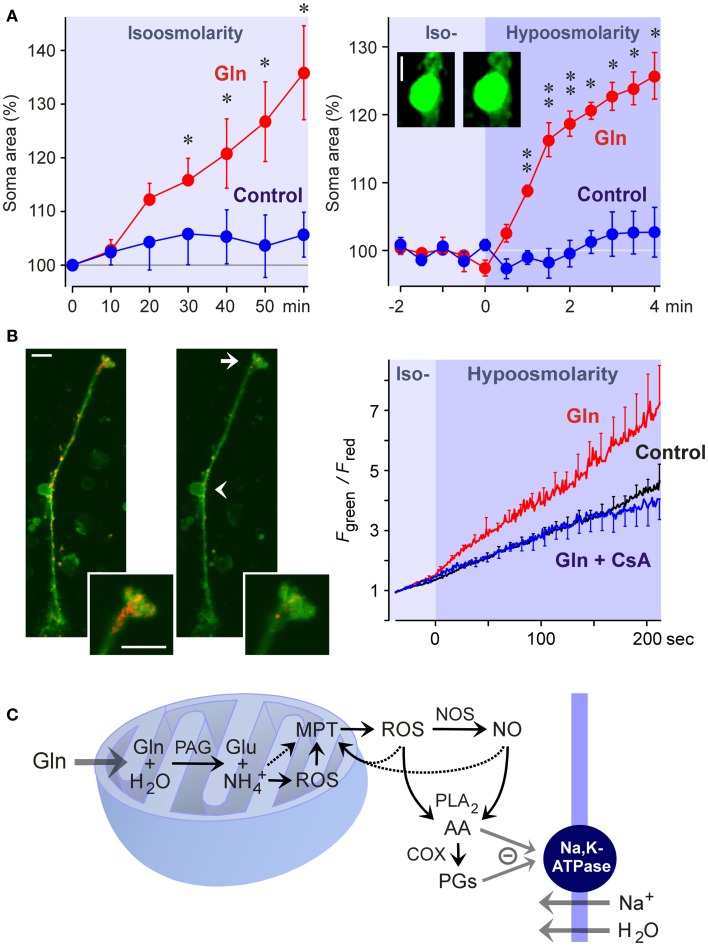
**Glutamine (Gln) induces mitochondrial dysfunction and Müller cell swelling**. Experiments were carried out in retinal slices **(A)** and isolated Müller cells **(B)** of the rat. **(A)** Glutamine (5 mM) in isoosmotic extracellular solution induced a delayed swelling of Müller cells after 10 min of exposure (*left*) whereas glutamine (5 mM) in hypoosmotic solution (60% of control osmolarity) induced a rapid (within 1 min) swelling of Müller cells (*right*). The cross-sectional area of Müller cell somata was recorded and is expressed in percent of the value obtained before superfusion of the slices with the solutions (100%). The *images* display original records of a Müller cell soma obtained before (*left*) and during (*right*) superfusion with the glutamine-containing hypoosmotic solution. Scale bar, 5 μm. Significant difference vs. control: *P < 0.05; **P < 0.01. **(B)** Glutamine induces a dissipation of the mitochondrial membrane potential in Müller cells. The potential was recorded from the endfeet of freshly isolated cells by using the mitochondria-selective potentiometric dye JC-1. Mitochondrial depolarization is indicated by an increase in the green-to-red fluorescence intensity ratio. *Left side:* Example of a JC-1-loaded cell which was recorded before (*left*) and during (*right*) superfusion of a hypoosmotic solution containing glutamine (5 mM). *Insets*, cell endfoot at higher magnification. Note the decrease in the red fluorescence. *Arrow*, cell endfoot. *Arrowhead*, cell soma. Bars, 10 μm. *Right:* Time-dependent change in the ratio of the green-to-red fluorescence of the JC-1 dye. The fluorescence was recorded in the absence and presence of glutamine (5 mM), before and during the transition of an isoosmotic to a hypoosmotic extracellular solution. Glutamine-induced a faster dissipation of the mitochondrial membrane potential as compared to control. The inhibitor of the mitochondrial permeability transition, cyclosporin A (CsA; 1 μM), prevented the glutamine-induced dissipation of the mitochondrial potential. **(C)** Hypothetical scheme of the mechanism of glutamine-induced Müller cell swelling. Cytosolic glutamine is transported into the mitochondria and is hydrolyzed there to glutamate (Glu) and ammonia (${\text{NH}}_{\text{4}}^{\text{+}}$) by the action of the phosphate-activated glutaminase (PAG). High ammonia levels stimulate the mitochondrial production of reactive oxygen species (ROS) and induce mitochondrial permeability transition (MPT) that leads to mitochondrial dysfunction, energy failure, and enhanced free radical production. Mitochondria-derived free radicals activate cytosolic enzymes that generate reactive oxygen and nitrogen species, e.g., nitric oxide synthases (NOS). These radicals stimulate the activity of phospholipases A_2_ (PLA_2_), that produce arachidonic acid (AA), lipoxygenases, and cyclooxygenases (COX) which generate prostaglandins (PGs). Arachidonic acid and prostaglandins inhibit the sodium-potassium-ATPase resulting in intracellular sodium overload, water influx, and cellular swelling. The energy failure due to mitochondrial dysfunction may contribute to the inhibition of the sodium-potassium-ATPase. With permission from Karl et al. (2011).

Liver diseases cause hyperammonemia associated with an increase in the cerebral ammonia to toxic levels (Swain et al., 1992; Tofteng et al., 2006). Hyperammonemia is a key factor in the pathogenesis of hepatic encephalo- and retinopathy (Reichenbach et al., 1995b; Albrecht and Norenberg, 2006). The major complication of fulminant hepatic failure is the development of brain edema characterized by swelling of astrocytes (Willard-Mack et al., 1996). In the neural retina, pathological alterations are found primarily in Müller cells and astrocytes; these alterations include cellular swelling (Figure 5A), mitochondrial dysfunction (Figure 5B), vacuolization, and necrosis (Reichenbach et al., 1995a; Karl et al., 2011). Ammonia-induced glial cell swelling depends on glutamine synthesis rather than on ammonia *per se* (Willard-Mack et al., 1996; Karl et al., 2011). The glutamine-induced swelling of Müller cells is accelerated under hypoosmotic conditions (Figure 5A) (Karl et al., 2011). Such conditions occur *in situ*; osmotic gradients across the glio-vascular interface are a result of ionic disbalances in the blood caused by, for example, hyponatremia due to liver disease and renal impairment.

The detoxification of excess ammonia is mediated by the formation of glutamine (Figure 3). Excess cytosolic glutamine is transported into the mitochondria where it is hydrolyzed to glutamate and ammonia by the phosphate-activated glutaminase (Figure 5C). High ammonia levels cause mitochondrial permeability transition (Figures 5B,C) and generation of free oxygen radicals (Albrecht and Norenberg, 2006; Karl et al., 2011). This results in mitochondrial dysfunction, energy failure, and in activation of cytosolic enzymes that generate oxygen and nitrogen radicals, e.g., nitric oxide synthases (Figure 5C) (Karl et al., 2011). Free radicals stimulate the production of inflammatory lipids including arachidonic acid and prostaglandins (Figure 5C) (Landino et al., 1996; Offer et al., 2005). These lipids inhibit the sodium-potassium-ATPase which results in intracellular sodium overload, water influx, and cellular swelling (Figure 5C) (Lees, 1991; Staub et al., 1994). The energy failure due to mitochondrial dysfunction and the high energy consumption of the glutamine synthetase reaction contribute to the inhibition of the sodium-potassium-ATPase. The decrease in glutathione synthesis (Figure 3) due to the enhanced glutamate consumption contributes to oxidative stress conditions (Reichenbach et al., 1999).

#### Production of glutathione

The uptake of GABA and glutamate by Müller cells links neuronal excitation with the defense against oxidative stress. Müller cells provide photoreceptors and neurons with an antioxidative environment (Bringmann et al., 2009). The major glia-derived antioxidant is reduced glutathione produced from glutamate, cysteine, and glycine (Figure 3) (Pow and Crook, 1995). Under normal conditions, retinal glutathione is concentrated in glial and horizontal cells (Pow and Crook, 1995; Schütte and Werner, 1998). In response to oxidative stress, glutathione is rapidly released from Müller cells and provided to neurons (Schütte and Werner, 1998) where it acts as cofactor of enzymes which remove toxic peroxides and regulate protein function through thiolation and dethiolation, such as glutathione peroxidase, reductase, transferase, and glutaredoxin.

The production of glutathione in Müller cells is critically dependent on the availability of extracellular glutamate and cystine (Figure 3) (Reichelt et al., 1997b). Inhibition or knockout of GLAST decrease the glutathione production (Reichelt et al., 1997b; Harada et al., 2007). Extracellular cystine is taken up mainly via the cystine-glutamate antiporter (Figure 3); inhibition of the antiporter results in a huge decrease in retinal glutathione (Kato et al., 1993). Inhibition of the antiporter can also result from an increase in extracellular glutamate (see above). Downregulation of the glutamine synthetase as occurring under pathological conditions (see above) results in a depletion of neuronal glutamate which also causes a decrease in retinal glutathione (Gionfriddo et al., 2009). Müller cells from aged animals contain reduced levels of glutathione; this is associated with mitochondrial damage, membrane depolarization, and reduced cell viability (Paasche et al., 2000).

#### Metabolic support of photoreceptors and neurons

The metabolization of glutamate in Müller cells is tightly coupled to the nutritive function of glia. In the retina, glucose uptake and metabolism occurs predominantly in the inner processes of Müller cells, localized to the inner plexiform and ganglion cell layers (Poitry-Yamate et al., 2013) where glutamatergic signaling occurs. Müller cells produce substrates for the oxidative metabolism of photoreceptors and neurons including glutamine, lactate, pyruvate, alanine, and α-ketoglutarate (Figure 3) (Poitry-Yamate et al., 1995; Tsacopoulos et al., 1997). These substrates are used by photoreceptors and neurons in periods of metabolic stress, e.g., in the dark. The production of lactate in Müller cells is stimulated by glutamate and ammonia (Poitry-Yamate et al., 1995; Poitry et al., 2000). Energy substrates are formed from glutamate also by other metabolic pathways, e.g., transamination of pyruvate (Figure 3) (Poitry et al., 2000). The conversion of glutamate into α-ketoglutarate is reduced in Müller cells of diabetic rats; this results in increased intracellular glutamate which impairs the glutamate uptake by Müller cells (Gowda et al., 2011).

## Concluding Remarks

Müller cells contribute to the removal of extracellular GABA and glutamate. The uptake and metabolization of the transmitters by Müller cells is part of the glutamate-glutamine cycle, and is linked to various other functions of the cells including the metabolic support of photoreceptors and neurons, the defense against oxidative stress, the shaping and termination of the synaptic neurotransmitter action, the release of gliotransmitters, and the detoxification of excess ammonia. Müller cells remove the bulk of extracellular glutamate (Rauen, 2000); this may be important for setting the signal-to-noise ratio of glutamatergic transmission.

Though research of the last two decades yielded a huge increase in our knowledge regarding the functional roles of Müller cells, there remain many open questions. A fundamental issue is the relative contribution of neuronal vs. glial GLTs in the shaping of excitatory synaptic responses. Likewise, the role of glial GATs in controlling retinal neurotransmission is presently unclear. The role of glial transporters in shaping of synaptic transmission may vary in dependence on the type of synapses. Photoreceptor terminals are suggested to directly remove glutamate from the synaptic cleft, with a minimal contribution of glial glutamate uptake (Hasegawa et al., 2006). On the other hand, recent evidence suggests a crucial role of GLAST-mediated glutamate uptake in the synaptic transmission within the outer plexiform layer (Levinger et al., 2012). Whether the opposing data reflect species differences remains to be clarified. It was suggested that Müller cells act as diffusion barriers for synaptically released glutamate in the outer plexiform layer that separates individual synapses from each other (Rauen et al., 1996). However, the recent finding of a glutamate spillover between cone photoreceptors (Szmajda and Devries, 2011) questions the existence of such a diffusion barrier. Glial transporters may play a more active role in shaping the excitatory responses in the inner retina (Matsui et al., 1999; Higgs and Lukasiewicz, 2002). More information concerning the functional roles of neuronal and glial GLTs at different retinal synapses is needed.

There are various other points which remain to be resolved. For example, it is not known to what extent Müller cells in retinas of birds and lower vertebrates take up GABA. The relative contribution of electrogenic vs. electroneutral transporters (Sarthy et al., 2005) in the glutamate uptake by Müller cells also remains an open issue. What are the specific functional roles of the different types and splice variants of GLTs in Müller cells? Which neuro- and gliotransmitters regulate the neurotransmitter recycling by Müller cells? How ammonia is taken up by the cells?

Dysregulation of Müller cell-provided glutamate recycling is a common phenomenon in retinopathies, and is associated with increased extracellular glutamate. The pathogenic mechanisms resulting in impaired glial glutamate uptake are incompletely understood. Inflammation and oxidative stress are major causative factors. The decrease in uptake might be caused by the downregulation/inactivation of the glutamine synthetase, by mitochondria-derived free radicals, which inactivate the transporter molecules, as well as by cell depolarization resulting from high extracellular potassium, energy failure due to mitochondrial dysfunction, downregulation/inactivation of potassium channels, and inhibition of the sodium-potassium-ATPase and GLTs through inflammatory lipid mediators. The depolarization of Müller cells may be strong enough to reverse the operation mode of GLTs resulting in an efflux of glutamate that contributes to excitotoxic neuronal damage. A significant portion of glial glutamate might be also released through the cystine-glutamate antiporter and vesicular exocytosis. Understanding the cellular mechanisms involved in the impairment of glial glutamate recycling may help to find novel targets for the development of neuroprotective agents. An increase in glutamine synthetase in Müller cells may represent such an approach which has been shown to protect against neuronal degeneration in the injured retinal tissue (Gorovits et al., 1997).

## Conflict of Interest Statement

The authors declare that the research was conducted in the absence of any commercial or financial relationships that could be construed as a potential conflict of interest.
